# Improved Membrane-Based Sensor Network for Reliable Gas Monitoring in the Subsurface

**DOI:** 10.3390/s121217058

**Published:** 2012-12-12

**Authors:** Detlef Lazik, Sebastian Ebert

**Affiliations:** Helmholtz-Centre for Environmental Research, T.-Lieser-Strasse 4, 06120 Halle, Germany; E-Mail: Sebastian.ebert@ufz.de

**Keywords:** gas, monitoring, soil, greenhouse gases, CO_2_, membrane, sensor

## Abstract

A conceptually improved sensor network to monitor the partial pressure of CO_2_ in different soil horizons was designed. Consisting of five membrane-based linear sensors (line-sensors) each with 10 m length, the set-up enables us to integrate over the locally fluctuating CO_2_ concentrations (typically lower 5%_vol_) up to the meter-scale gaining valuable concentration means with a repetition time of about 1 min. Preparatory tests in the laboratory resulted in a unexpected highly increased accuracy of better than 0.03%_vol_ with respect to the previously published 0.08%_vol_. Thereby, the statistical uncertainties (standard deviations) of the line-sensors and the reference sensor (nondispersive infrared CO_2_-sensor) were close to each other. Whereas the uncertainty of the reference increases with the measurement value, the line-sensors show an inverse uncertainty trend resulting in a comparatively enhanced accuracy for concentrations >1%_vol_. Furthermore, a method for *in situ* maintenance was developed, enabling a proof of sensor quality and its effective calibration without demounting the line-sensors from the soil which would disturb the established structures and ongoing processes.

## 1. Introduction

Gases such as carbon dioxide (CO_2_), oxygen (O_2_) and methane (CH_4_) are important for various environmental and technical processes and have to be monitored for understanding and controlling certain processes. Furthermore, gases from natural subsurface sources, hydrogen (H_2_) or CH_4_ from gas leaks in repositories and pipelines or released by gas fracking and CO_2_ leaking from geological sequestration can impact on carbon accounting and/or may present serious risks for humans, animals and plants. To extend the lead time for managing these types of risks, a reliable baseline monitoring of gases within the subsurface is required to characterize gas concentrations before these enter the biosphere. To determine the level of concentration, the specific conditions of measurement have to be considered, such as phase composition (solid, liquid and/or gaseous phases) within the observed system and its variability which could restrict the applicability of particular measurement techniques.

Additionally, in large-scale and often heterogeneous systems such as bioreactors, landfills, aquifers or soils, local information on the gas concentration could be insufficient to determine or control the ongoing processes at a relevant scale [1]. Here it could be necessary to integrate over the locally fluctuating concentrations or fluxes [2]. With the exception of optical open path detectors however, gas measurement systems are typically based on the direct interaction with the gas molecules within a spatially restricted sensitive area or a technical support volume which are mostly designed for local measurements. Open path sensors on the other hand are only applicable in gaseous systems, e.g., in the atmosphere.

To expand this valuable integrating approach over heterogeneous natural systems such as soils, aquifers, surface water bodies or geotechnical/technical situations (repositories, reactors, *etc.*) mechanically robust gas sensors are required which: (a) can gather reliable information from large areas; (b) work efficiently within the subsurface; (c) are insensitive to changing phase saturations and (d) show an acceptable response time.

The accessibility and possibility of the proof of sensor quality forms a second vital issue for the reliability of data gained. Airborne measurement systems can be maintained face to face which allows the operational reliability to be easily checked, meaning that the measurement system can be recalibrated without technical limitations or serious influences on its environment.

Measurement systems which are running in the subsurface have to fulfill comparable maintenance properties if they are to be acceptable for practical utilization. But an installed sensor normally has to be removed from its observation position within the subsurface for checking and calibration. This increases effort, reduces reliability and influences the structure and ongoing processes within the observed space, particularly for long term monitoring required in geological sequestration operations.

Up until now, both issues—the noninvasive, integrating gas measurement in the subsurface and the *in situ* maintenance of the measurement systems—have posed ambitious challenges to sensor technology. Additionally, as demonstrated in current public discussion, the lack of suitable safety techniques could hamper the acceptance of new environmental technologies such as the geological storage of CO_2_ or the fracking of shale gas formations.

A new membrane-based measurement technology for gases was introduced in [3] for future application within the environment, e.g., in the subsurface. The operation of a membrane-based tubular sensor (line-sensor) is based on the diffusive fluxes of gases through the wall of a gas-selective membrane. Two steps form a measurement cycle. First (the initialization step), the fluxes have to adjust. Therefore, the inner wall of the membrane tube has to be flushed (conductive conditioning) with a reference gas of known composition, e.g., air, to establish dynamic equilibrium (steady-state fluxes). The concentration-specific steady-state fluxes superpose with each other and correspond in their result with a diffusive change of the total mole number within the tube (sensor chamber). If at *t* = *t*_0_ in the second step of the measurement cycle (measurement step) this chamber is closed, the internal gas pressure *p* will be characteristically changed. This change can be related in the form of a linear dependency to the partial pressures $p_{k}^{a}$ of the outer space:

(1) $${\frac{\mathrm{dp}}{\mathrm{dt}}|}_{t\to t_{0}}=a_{1}=gP_{s}\sum_{k=1}^{n}f_{\mathrm{ks}}(p_{k}^{a}-p_{k}^{i}),\;\;\;\;\;f_{\mathrm{ks}}=P_{k}/P_{s}$$

where *p* [mbar] is pressure, *t* [s] is time, *a*_1_ [mbar/s] is pressure change at *t* → *t*_0_, *g* [*m*^−2^] is geometry factor of the sensor, *P_s_* [m^2^/s] is membrane permeability for the gas index “s”, *f_ks_* is selectivity of the gas “*k*” with respect to the gas “*s*”, $p_{k}^{a}$, $p_{k}^{i}$ [mbar] are the partial pressures of the gas “*k*” within the gaseous spaces outside (index “*a*”) and inside (index “*i*”) the sensor and *n* is the number of gases.

For the measurement of a single gas within a given gaseous matrix (e.g., air, soil air), Equation (1) was simplified, resulting in a linear dependency of *a*_1_ to the gas of interest and additionally in a reduced complexity of the sensor construction. This type of sensor has to be calibrated against different concentrations of that gas. Within a measurement comparison, an accuracy of 800 ppm could be demonstrated for the observation of CO_2_ in which the calibrated line-sensors were run within a lysimeter filled with a dry soil [3].

Deployment in soils could be an important application field for measurement technology. Due to its large storage capacity for carbon, soils contribute significantly as a source and sink for atmospheric greenhouse gases [4]. A detailed understanding of the underlying processes could be helpful for a sustainable handling of this valuable resource [5] and therefore long-term field studies are necessary to study CO_2_ dynamics in soils.

Typically, the CO_2_ concentration ranges up to 5%_vol_. Here the CO_2_ is produced through root respiration, microbial respiration and the oxidation of organic matter and varies according to temperature, moisture, soil type, cultivation, vegetation cover and is additionally dependent on temporal changes in land use. Temporal highly resolved point measurements performed, e.g., with the aid of solid-state sensors [6], allow valuable insights into the natural process dynamics at a local scale.

In order to integrate with a sufficient temporal resolution up to the meter-scale over the locally fluctuating CO_2_ concentrations, a conceptually improved monitoring set-up was designed based on the developed line-sensor technology. An evaluation was performed of the set-up prior to its prospective deployment in soil. Based on the results of this evaluation the paper focus on two aspects: the increase in accuracy with respect to the previous published state-of-the-art [3] and, a new issue of this gas sensor technology: the *in situ* maintenance of line-sensors.

## 2. Improved Sensor Set-Up and Calibration Methods

**Figure 1.**
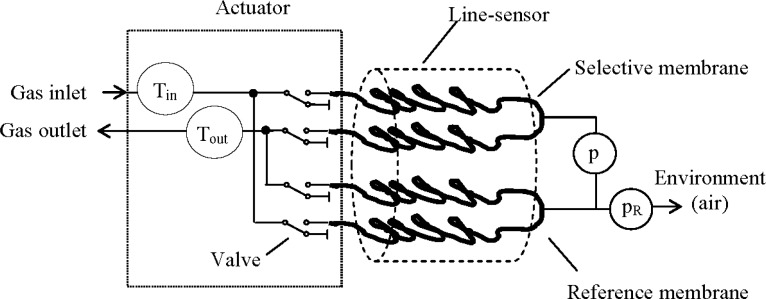
Measurement set-up: for flushing the line-sensor, an actuating unit, containing sensors (T_in_, T_out_) for registration of the reference gas temperature and four valves, connects the line-sensor with a gas supply. The line-sensor is composed of a selective membrane and a reference membrane. The concentration sensitive pressure change *p*(*t*) and the reference pressure *p_R_*(*t*) were measured at the half-length of the flow paths within the sensor tubes.

In cooperation with the manufacturer MeGaSen (Potsdam, Germany, www.megasen.com) an improved prototype set-up was developed for the monitoring of natural soil CO_2_ consisting of five line-sensors (S1–S5) which were driven by actuating units. To perform the measurement cycle each unit (Figure 1) contains four valves for flushing a line-sensor by a reference gas (initialization step) and sealing it (measurement step). Here two valves are connected to the gas selective membrane and the other two to the reference membrane with a common gas in- and outlet, respectively. Integrated temperature sensors observe the reference gas temperature at both line-sensor ends.

Actuators and sensors are connected with a PC-based control unit. Two membrane pumps were used for flushing the line-sensors with a reference gas. The line-sensors were made with a 10 m length of polydimethylsiloxane (PDMS) tubing (gas selective membrane) and polyurethane tubing (reference membrane). The tube bundle was protected against mechanical damage by a meshed hose made from 0.35 mm polyethylene fiber. Pressure sensors were coupled on one side of the line-sensor and the actuating unit was connected at the opposite side. With respect to a possible construction distance between the monitoring site and the position of the control station, color-coded spacer tubes with gas-tight walls (polyurethane, *L* = 1 m) were added at both ends of the line-sensors. All tubular membranes have an inner radius *R_i_* = 0.7 mm and an outer radius *R_o_* = 1.8 mm.

The line-sensors were run sequentially: while the pressure evolution within one line-sensor was analyzed, the other line-sensors were flushed simultaneously by the reference gas. The time span for a measurement step was adjusted to 5 s; the repetition time of the measurement with a particular line-sensor was 73 s.

### 2.1. Compensation of Pressure Dependencies

(I) According to Bernoulli’s equation a linear drop (<50 mbar) of the gas pressure along the line-sensor should be expected during flushing with the reference gas (initialization step). Thus, the partial pressures of the gas components also drop, forming slightly changing dynamic equilibriums over the membrane wall. As a result, a weak position dependency of the sensor response has to be expected. In compensation, each membrane was placed twofold within a line-sensor (see Figure 1) and coupled at one end with each other, resulting in a constant pressure mean at each position of the line-sensor. This mean pressure is given by the reference sensor *p_R_* which is positioned at the half length of the total gas flow path.

(II) In the previous design [3], the reference gas was flushed with the pressure *p_in_* into the line-sensor inlet and escapes from its outlet against the air pressure *p_atm_* causing the mean overpressure *p_R_=* (*p_in_* − *p_atm_*)/2. This overpressure contributes to the measurement result in *a*_1_, enhances its error rate and has to be considered within the line-sensor calibration.

In compensation, the line-sensors have to be run in a mean pressure level which is close to the outer gas pressure (*p_R_* → 0). Two membrane pumps were therefore connected with the actuating units: one pump compresses the reference gas into the line-sensor and the other sucks it out. The pumping rates were roughly adjusted to minimize the pressure offset *p_R_*.

### 2.2. Compensation of the Temperature Dependency

The gas flow through a membrane is determined by its material parameters: solubility S [-] and diffusion coefficient D [m^2^/s]. For symmetrical membranes, both parameters form the permeability constant *P = S·D* in Equation (1). All three material parameters are correlated exponentially with the temperature [7]. According to the simplified sensor theory (Equation (8) in [3]):

(2) $$a_{1}(t)=gP_{x}\left(\Delta p_{x}^{a}(t)\left(1-\frac{1}{p^{a}}\sum_{k}f_{\mathrm{kx}}p_{k}^{a}(t_{0})\right)+\sum_{k}f_{\mathrm{kx}}(p_{k}^{a}(t_{0})-p_{k}^{i}(t_{0}))\right)$$

the permeability $P_{x}\propto \text{exp}(-E_{x}^{p}/\mathrm{RT})$ shows a strong temperature dependency, where $E_{x}^{p}$ [kJ/mol] is the gas specific activation energy of permeation of the measured gas “*x*”, R = 8.3144 J/mol/K is the gas constant and T [K] the temperature. The temperature dependency of the selectivity’s: $f_{\mathrm{kx}}\propto \text{exp}(\Delta E_{\mathrm{kx}}^{p}/\mathrm{RT})$, ${\Delta E}_{\mathrm{kx}}^{p}=E_{k}^{p}-E_{x}^{p}$ within the sums of Equation (2) are comparatively smaller and will be neglected in a first approximation. Subsequently, a pressure change measurement for temperature *T* could be normalized with respect to the reference temperature *T*_0_, by:

(3) $$a_{1}(T_{0})\approx a_{1}(T)\text{exp}\left(\frac{E_{x}^{p}}{R}\left(\frac{1}{T_{0}}-\frac{1}{T}\right)\right)$$

### 2.3. Calibration Methods

Notionally, line-sensors can be calibrated based on sensor theory using the known sensor geometry and permeability constants from the literature [8]. Practical experience demonstrates a higher accuracy for the experimentally calibrated line-sensor and therefore two methods will be applied for calibration:

**Direct calibration:** For direct calibration (applied in [3,9]) the line-sensors and a reference sensor have to be installed within a suitable calibration set-up. In that they have to be exposed to different gas mixtures. The relationship between the concentration $p_{{\mathrm{CO}}_{2}}^{a}$ and the sensor response can be approximated by the linear dependency:

(4) $$p_{\mathrm{CO}2}^{a}=(k_{1}\pm {\delta k}_{1})a_{1}+(k_{0}\pm {\delta k}_{0})$$

where *a*_1_ [mbar/s] is pressure change, *k*_1_ [%_Vol_ s/mbar] is slope, *k*_0_ [%_Vol_] is offset and δ*k*_1_, δ*k*_0_ are the standard deviations of calibration constancies. For a well-adjusted mean reference gas pressure (ideal working point) within the sensor tube, the offset *k*_0_ has to be small.

The method enables the determination of the true sensor response. However, the method requires homogeneous distributed well-known gas concentrations within the supporting space of the line-sensor. For this purpose, the line-sensor has to be demounted from the observation object.

**Inverse calibration:** During inverse calibration, the known individual gas mixtures $p_{{\mathrm{CO}}_{2}}^{i}$ were flushed through the line-sensor itself forming dynamic equilibriums against the unknown background concentration $p_{{\mathrm{CO}}_{2}}^{a}$. Assuming a linear dependency between the concentration difference between both faces of the line-sensor and its response *a*_1_:

(5) $$p_{{\mathrm{CO}}_{2}}^{i}=(k_{1}^{\mathrm{inv}}\pm \delta k_{1}^{\mathrm{inv}})a_{1}+(k_{0}^{\mathrm{inv}}\pm \delta k_{0}^{\mathrm{inv}})+p_{{\mathrm{CO}}_{2}}^{a}$$

where the index “inv” refers to “inverse” and an adjusted ideal working point ( $k_{0}^{\mathrm{inv}}\to 0$) of the line-sensor, the remaining offset should represent the unknown background concentration $p_{{\mathrm{CO}}_{2}}^{a}$ during calibration. This enables the calibration of the line-sensor without having to demount it from the observation object. Due to the cylindrical sensor geometry, one expects a difference in the slopes from both methods, but this difference should be related to geometrical properties of the line-sensor.

## 3. Experiments

To analyze the accuracy of the sensory system and verify the applicability of the inverse calibration method, different experiments were performed in the laboratory. The line-sensors were placed within a closed 0.1 m³ vessel together with the reference. The vessel was equipped with a gas entry and a gas outlet. The gas entry was connected with a calibrated mixing station formed by two mass flow controllers (MFC, Bürkert Fluid Control Systems, Ingelfingen, Germany) for CO_2_ and air. A fan was added to enhance the homogeneity of the gas composition within the vessel. As a reference an NDIR-sensor for CO_2_ (CARBOCAP^™^-GMP221, VAISALA, Hamburg, Germany): measurement range 0 to 10%_Vol_, precision <± (0.0002 + 0.02 pCO_2_) was installed in the center of the vessel. For direct calibration and monitoring tests the vessel was flushed by defined CO_2_–air mixtures from top to bottom. Air was flushed through the line-sensors as a reference gas. For inverse calibration, the vessel was flushed with air in a first experiment and with air mixed with a constant CO_2_ background (second experiment). Known CO_2_–air mixtures were flushed through the line-sensors. The CO_2_ concentrations within the gas mixtures were analyzed simultaneously by both the reference and the installed line-sensors.

## 4. Results and Discussion

### 4.1. Compensation of Pressure Dependencies

**Figure 2.**
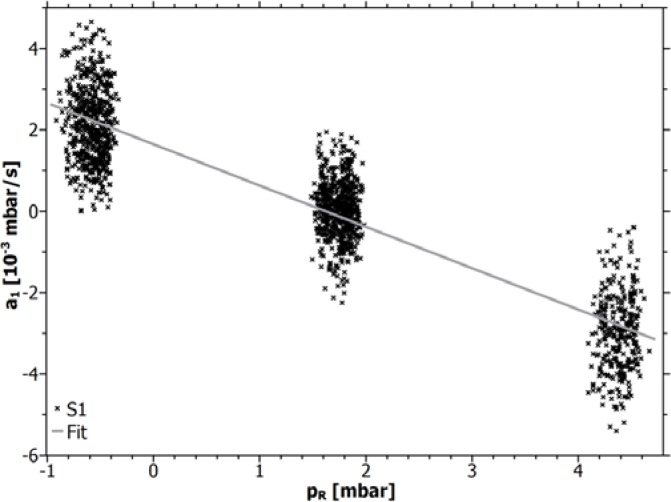
Influence of the pressure offset on the pressure change *a*_1_ for line-sensor S1.

Figure 2 shows the pressure dependency on the sensor response: *a*_1_ = *c*_0_ + *c*_1_·*p_R_* for an equivalent gas composition (air) at both faces of line-sensor S1. The fitted constants *c*_1_ [s^−1^] and *c*_0_ [mbar/s] are shown for all line-sensors in Table 1.

An overpressure within the line-sensors corresponds with a diffusion direction from its inner to its outer membrane face. The resulting sensor response will be negative. Whereas *c*_0_ = 0 can be expected for a planar membrane, diffusion through a cylindrical membrane corresponds to *c*_0_ > 0. As shown in Figure 2, the pressure influence disappears at a pressure offset of about 1.6 mbar. It is obvious that for sensor calibration an adjustment of the pressure offset defining the working point of the line-sensor has to be performed and that fluctuations around this working point will contribute to the calibration error. For the ideal working point at *p_R_*(*a*_1_ = 0) = 1.6 mbar the sensor response corresponds exclusively to the concentration differences of the gases at both faces of a line-sensor. Using the simple set of membrane pumps, the working point varied accidentally between −0.5 to 1.3 mbar. Assuming that the sensor-specific pressure dependency *β* = *c*_0_ + *c*_1_·*p_R_*(*t*) should in an initial approximation be independent of the gas composition, the influence of the remaining pressure offset on the sensor response can be reduced by *a*_1_(*p_R_*) – *β* approximating the ideal working point.

### 4.2. Calibration Comparison

**Figure 3.**
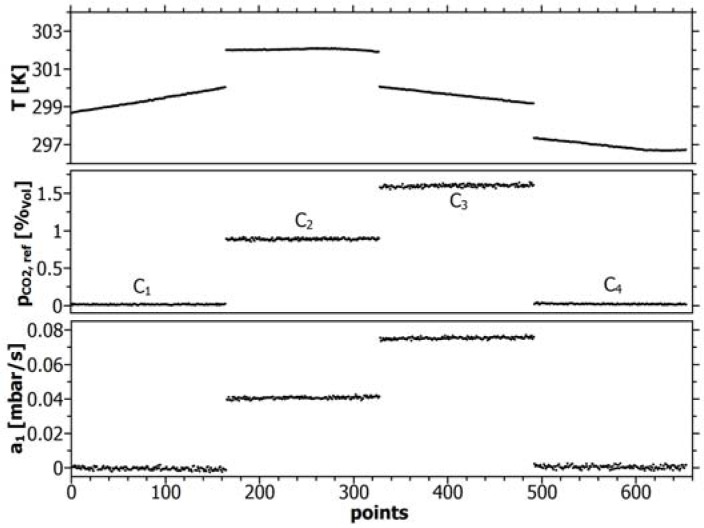
CO_2_-concentration steps (C_1_–C_4_) for calibration, response *a*_1_ of line-sensor S1 and temperature T during calibration.

For a comparison of direct and inverse calibration, only adjusted concentration plateaus were evaluated to avoid misinterpretation due to local concentration differences within the calibration set-up. This step-like concentration distribution within the vessel, the corresponding (offset-pressure reduced) sensor response *a*_1_ and the gas temperature are shown in Figure 3.

**Figure 4.**
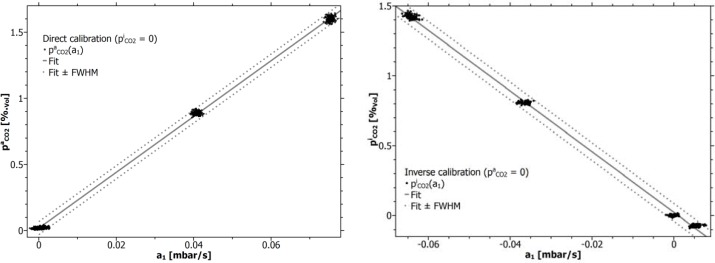
Results from direct (**left**) and inverse calibration (**right**). Mirror inverted slopes of the response functions result from the different orientations of the diffusive fluxes through the membrane wall. Dashed lines limit the 2.35-fold of the standard deviation (FWHM is full widths at half maximum).

For inverse calibration, the concentration sequence according to Figure 3 was used for flushing the line-sensors during the conditioning step. Figure 4(left) and Table 2 show the results for direct and Figure 4(right) and Table 3 for inverse calibration, respectively. Both calibrations were run against air. The pressure offsets (working point) ranged between (−0.53 ± 0.44) mbar for direct and (1.26 ± 0.15) mbar for inverse calibration.

The figure (Figure 4) and the tables (Tables 2 and 3) demonstrate a perfect match between the line-sensors and the reference with correlation coefficients better 0.998 for all fits.

The remaining small offsets *k*_0_ document that the performed adjustment of the ideal working point still has to be improved. This necessitates a controlled pump set and will be tested in the future. Irrespective of the individual calibration method, the accuracy *δp*_*CO*_2__ of the approximated data documents a considerable gain in accuracy with respect to the previously reported 800 ppm [3].

A comparison of *k*_1_ and $k_{1}^{\mathrm{inv}}$ (Tables 2 and 3), by its averages results in: $-k_{1}/k_{1}^{\mathrm{inv}}$ = 0.959. However, almost the same value can be calculated through the geometrical argument *α =* ln(1 + *w/R_i_*) = 0.944, where *w = R*_0_ − *R_i_* is the wall thickness of the membrane. Containing in the geometry factor of a line-sensor [9], this argument marks the opening of the space for gas diffusion within the wall of a cylindrical membrane. This correspondence was also found for line-sensors with *R*_i_ = 0.8 mm and w = 0.4 mm where $k_{1}/k_{1}^{\mathrm{inv}}$ = *α =* 0.405 [10].

The transformation from inverse back to the direct calibrated slope is therefore assumed to be related to the geometrical property: $k_{1}=\alpha \cdot k_{1}^{\mathrm{inv}}$. When applying this transformation, the slope is *k*_1_ = 20.75%_vol_ s/mbar, resulting in a deviation of about 2% from the average calculated from the direct calibrated slopes (Table 2).

### 4.3. Comparison and Enhancement of Accuracy

**Figure 5.**
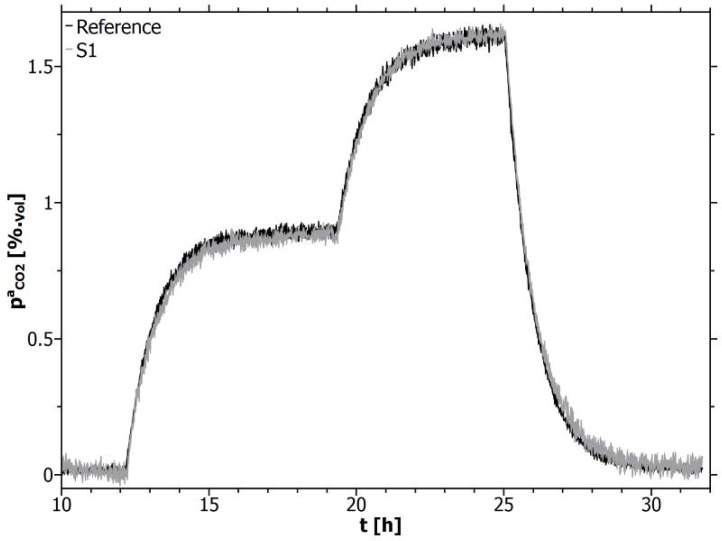
Monitoring comparison between the line-sensor S1 and the reference shown for a dynamic evolution of the CO_2_ concentration within the calibration vessel. The accuracy of the reference exceeds that of the line-sensor for small concentrations.

Figure 5 shows a measurement comparison of the direct calibrated line-sensors (grey) with the reference (black) for a dynamic developing CO_2_ concentration within the calibration vessel.

**Figure 6.**
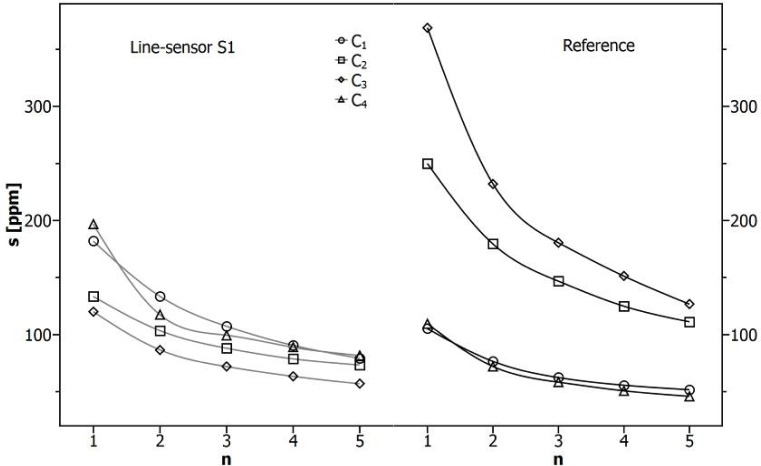
Concentration dependent standard deviations (s) resulting from smoothing with a moving average over n elements. For the higher concentrations C_2_ and C_3_ the line-sensor S1 (**left**) shows a lower standard deviation with respect to the reference (**right**).

The figure demonstrates a sufficient fast response behavior to follow the concentration evolution. In particular for higher concentrations, a greater degree of accuracy is shown with respect to the reference. This effect is highlighted in more detail in Figure 6.

In Figure 6, a comparison is shown between the independently estimated standard deviations of the sensor signals for line-sensor S1 (left) and the reference sensor (right) within the plateau-concentrations C_1_−C_4_ marked in Figure 3. The obtained data of all line-sensors and the reference are shown in Table 4.

Furthermore, we compare the impact of a moving average with n elements for minimizing the deviations. To extract independent standard deviations s [ppm] for the line-sensor response, those of *a*_1_ were calculated within the respective data ranges and transformed using Equation (4) and the fit constants from Table 2. In the same manner, we applied the moving average on the records of *a*_1_, calculated the remaining standard deviations and transformed the results. Synchronized data points were selected to estimate the corresponding standard deviation of the reference.

Figure 6 demonstrates that the line-sensor S1 shows a lower standard deviation with respect to the reference for the concentrations C_2_ and C_3_. Although the deviation of the reference increases with the concentration, the deviation of S1 shows the opposite trend. This tendency will be confirmed in the middle by Table 4 contradicted only by the high deviation of line-sensor S2 for concentration C_3_.

It can therefore be assumed that higher concentrations which could also exceed the concentration range considered in this study could be analyzed with a greater degree of accuracy. Furthermore, a high gain in accuracy (s < 100 ppm) can be realized by smoothing over 4 to 5 elements corresponding with a temporal resolution of 5 to 6 min which is sufficient for observing the CO_2_-dynamics in a soil.

### 4.4. Compensation of the Temperature Dependency

The gas temperature was analyzed at the line-sensor inlet and its outlet (see Figure 1). Assuming a fast equilibration between the reference gas and the membrane material, the outlet temperature was tested to reduce the temperature dependency according to Equation (3).

**Figure 7.**
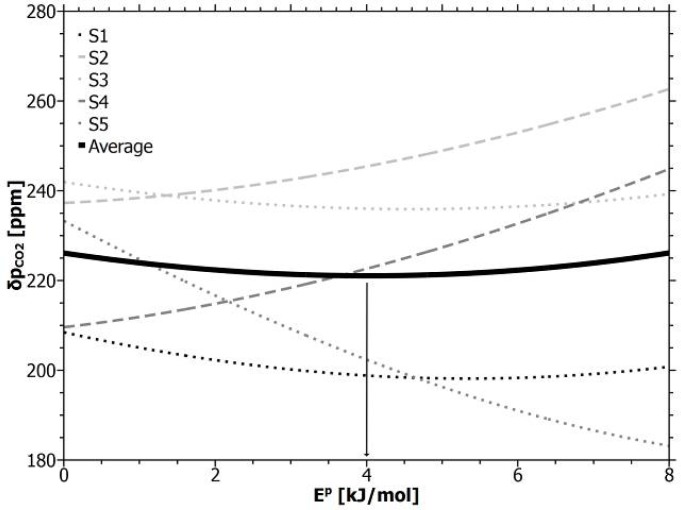
Accuracy of temperature-compensated line-sensors S1−S5 in dependence to the simulated activation energy *E^p^*. For *E^p^* = 0 the shown accuracy corresponds with the data in Table 2. The arrow marks the minimum of the average over individual accuracy dependencies.

A reference temperature (300 K) was chosen close to room temperature ranging over 5 K between 297 and 302 K during the experiment. Our own permeation experiments result in an activation energy $E_{\mathrm{CO}2}^{p}$ = (3.9 ± 0.4) kJ/mol for a pure PDMS-membrane. The activation energy could however possibly vary for commercially available PDMS-tubes, for example depending on additives, fillers, impurities or the production process. This activation energy was therefore varied within the range *E^p^* ≤ 8 kJ/mol and reduced data sets were generated from the measured sensor response *a*_1_ of the direct calibration run (shown in Figure 3). The temperature-reduced sensor responses were calibrated against the reference data. The resulting accuracies were compared in their dependence to the simulated activation energy in Figure 7. The figure shows an individual behavior of the line-sensors and a weak minimum of the averaged accuracy (thick line). The energy (about 4 kJ/mol) at the minimum position corresponds with the experimentally estimated activation energy for PDMS which can be interpreted as a sign for the applicability of Equation (3), relating the temperature dependency to a material constant. The temperature compensation, however, does not produce any considerable improvement in accuracy (in the mean about 10 ppm) and therefore temperature compensation remains negligible within a range of several Kelvin.

### 4.5. Calibration against an Unknown Background Concentration of CO_2_

The final questions are: (I) is a line-sensor calibrated inversely against air able to quantify an outer background concentration (*i.e.*, calibration before installation) and (II) is its calibration within a dynamical system such as soil possible (*i.e.*, calibration after installation)? To respond to these questions, the calibration vessel was flushed with air which was mixed with some CO_2_ resulting in a mean concentration of around $p_{{\mathrm{CO}}_{2}}^{a}$ = (0.872 ± 0.029)%_vol_. The concentration within the vessel was varied to simulate a dynamic gas behavior and was analyzed by line-sensors and a reference.

Again, reference gases with the concentrations C_1_–C_4_ according to Figure 3 were used for the conditioning step in which the offset pressure within the sensor tubes remained at a level of around *p_R_* = (−0.29 ± 0.177) mbar.

The line-sensor responses were transformed using Equation (5) and the constants ( $k_{0}^{\mathrm{inv}}$, $k_{1}^{\mathrm{inv}}$) from Table 3 to answer the first question (Test I). The unknown background concentration $p_{{\mathrm{CO}}_{2}}^{a}$ has to result from averaging of the calculated data. With regard to the second question, the same line-sensor responses were then once more inversely calibrated—in this case against the dynamic background (Test II).

**Figure 8.**
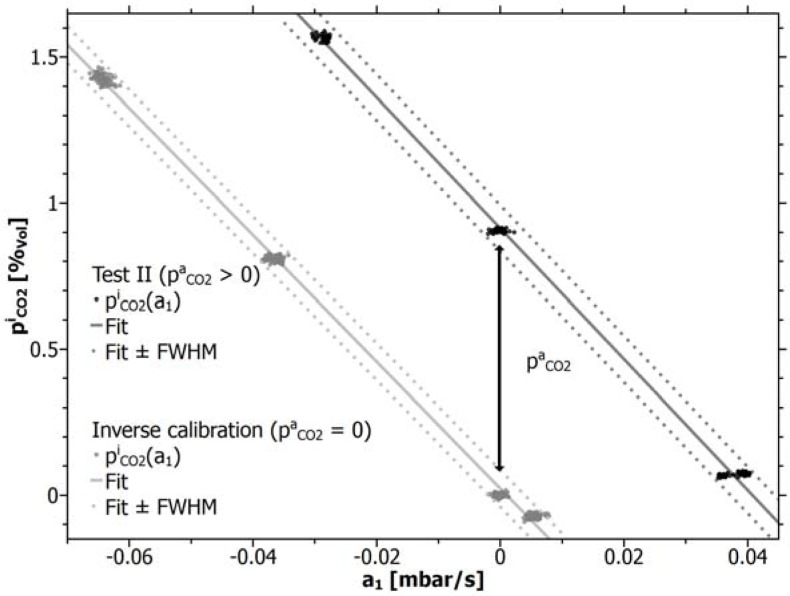
Comparison of the inverse calibrations for line-sensor 1: against air (light line) and against the unknown background concentration $p_{{\mathrm{CO}}_{2}}^{a}$ (dark line). The vertical distance between the offset-reduced lines represents $p_{{\mathrm{CO}}_{2}}^{a}$.

Figure 8 shows the inverse calibration of line-sensor 1 for air (light line, already shown in Figure 4) and against the unknown $p_{{\mathrm{CO}}_{2}}^{a}$ (dark line, Test II). Table 5 contains the respective calibration constants for all line-sensors for Test II. Both lines in Figure 8 appear to be parallel to each other. This implies that, independent of the amount of $p_{{\mathrm{CO}}_{2}}^{a}$ both lines have to show the same slope for unaltered material properties of the sensor membrane. Vice versa, a (long-term) change of material properties has to be indicated by a shift in the slope. According to Equation (5), also for altering slopes, it follows $p_{{\mathrm{CO}}_{2}}^{i}=k_{0}^{\mathrm{inv}}+p_{{\mathrm{CO}}_{2}}^{a}$ at *a*_1_ = 0 as the difference between both calibration lines. Here the unknown $k_{0}^{\mathrm{inv}}$ should be similar to the remaining offset constant within the calibration against air and will be reduced by the corresponding offset from Table 3.

The accuracy *δp*_*CO*_2__ shown in Table 5 appears to be reduced with respect to the accuracy for inverse calibration against air (Table 3). We assume that this effect corresponds to fluctuations of the background concentration, but also concentration differences between the individual supporting volumes of the line-sensors and the reference will contribute to the effect, caused by a non-ideal mixing and concentration gradients within the calibration vessel.

**Figure 9.**
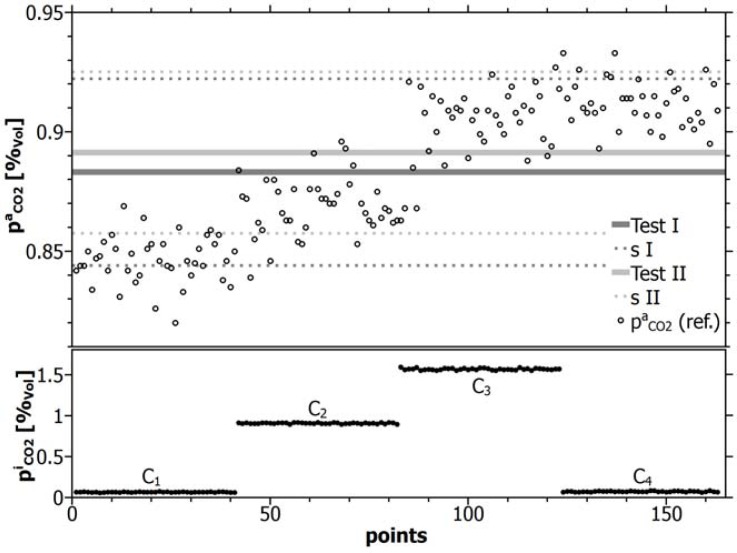
Upper diagram: background concentration within the vessel (circles) observed directly by the reference and determined during the course of inverse calibration tests (lines) for line-sensor 1. The dark line represents Test I and the light line Test II. Dotted lines show the standard deviations. The lower diagram shows the respective CO_2_ concentrations (C_1_–C_4_) of the reference gas for inverse calibration.

Figure 9 shows the background concentrations determined by line-sensor 1 in the course of its calibration during Test I (dark lines) and Test II (light lines) and the direct observation of the reference for the concentration evolution within the vessel. Table 6 contains the mean background concentrations determined by the individual line-sensors.

As demonstrated by Figure 9 and Table 6 both tests (I and II) enable the indirect estimation of the background concentration with a high accuracy within a dynamic gas system. The remaining small difference between both concentration means remained within a range of 100 ppm.

This demonstrates that a pre-calibrated line-sensor (Test I) is able to determine an unknown outer background concentration. Moreover, it could also be demonstrated that it is possible to calibrate an installed membrane-based line-sensors without the necessity of demounting (Test II). Such inverse calibration considers the ambient conditions, e.g., the ambient gas composition, physical soil properties and the temperature within the soil and enables therefore a reliable gas monitoring in a high accuracy over a long period.

The comparatively high uncertainty *δp*_*CO*_2__ ≤ 0.043%_vol_ of the mean background concentration and the asymmetry of the sensor response around the estimated means (see in Figure 9) can be attributed mainly to the real evolution of the background concentration and can be used as criteria for a further improvement of calibration.

## 5. Conclusions

A membrane-based line-sensor network was developed for the reliable permanent CO_2_ monitoring in soil. With five line-sensors measuring 10 m in length the improved set-up enables integration over the locally fluctuating CO_2_ concentration up to the meter scale with a temporal resolution of around 1 min.

The network was evaluated prior to its prospective deployment in soil. Thereby, a considerable increase in accuracy could be established to less than 300 ppm compared with the previous figure of 800 ppm. Here, the line-sensors show a distinctly smaller signal deviation for higher concentrations with respect to the optical reference sensor. Signal smoothing performing by a moving average over 4–5 elements results in a standard deviation of below 100 ppm.

The improvement in accuracy was achieved through an advanced conceptual/technical set-up and an adjustment of the reference gas pressure (working point) defining the boundary conditions for the diffusive gas fluxes through the membrane wall of the sensor which form the sensor response.

Furthermore, a method was developed to compensate for possible temperature effects based on material properties of the sensor membrane. However, a first application within a small temperature range of around 5 K shows only a negligible dependency. This result can be attributed to the reference-based measurement principle in which the reference gas acts as an internal standard.

In addition, the result implies that the influence of slight local temperature variations at whose mean within the supporting area of a line-sensor also remains negligible. This is important for the measurement in both natural and geotechnical system in which such local temperature variations could result for various reasons including radiation, clouding, biological activity, water content, evaporation or condensation, or a movement of gases or liquids through the supporting area of the sensor.

An ambitious challenge for gas observation systems which are operated stationary within the subsurface is the confirmation of functional efficiency. The higher the expectations are for the reliability of the collected data, the more need there is for proof. For this purpose, a method for *in situ* maintenance has to be available to calibrate the sensors. For calibration, the gas concentration has to be known within the supporting volume of a sensor (direct calibration). But this is a-priori not available for a heterogeneous natural system such as a soil. Hence, an unknown concentration has to be considered and determined within the calibration procedure. For this purpose we developed an inverse calibration method. It enables the effective calibration of the line-sensor without the necessity of demounting the sensor from the soil which would otherwise disturb the established structures and ongoing processes. The inverse calibration method was successfully demonstrated against an unknown background concentration and could be validated using an independent reference sensor. Allowing for the individual supporting volumes of the different sensors the measurement comparison was performed within a closed vessel. Moreover, a transformation was found between direct and inverse calibration based on the geometrical properties of a line-sensor.

A crucial issue of the deployment of line-sensors within the subsurface is formed by the membrane stability against chemical and biological attack. This issue has to be investigated in prospective field studies. However, a key to assess the operational availability of a line-sensor is the information about its sensitivity and therefore, the inverse sensor calibration forms also a powerful tool to evaluate the lifetime of the sensor within whose particular environment.

The deployment of line-sensors in the field is on an early stage. We assume line-sensors can be developed covering a large range from a few decimeters up to about 100 to 200 m. First successful field experiments were performed with 40 m line-sensors which were installed in two depths (20 cm, 30 cm) below the soil surface on top of each other. The results will be reported in a forthcoming paper.

## Figures and Tables

**Table 1.** Fit constants of the sensor response *α*_1_ ± *δα*_1_ = *c*_0_ ± *δc*_0_ + (*c*_1_ ± *δc*_1_) *p_R_* with their dependence on the reference gas pressure for equivalent gas composition at both faces of the line-sensors.

**Sensor**	***c*_0_ × 10^3^ [mbar/s]**	***δc*_0_ × 10^3^ [mbar/s]**	***c*_1_ × 10^3^ [*s*^−1^]**	***δc*_1_ × 10^3^ [*s*^−1^]**	***δα*_1_ × 10^3^ [mbar/s]**
S1	1.649	0.028	−1.016	0.012	0.88
S2	2.297	0.030	−0.998	0.013	0.96
S3	1.991	0.030	−1.046	0.013	0.95
S4	1.510	0.028	−0.999	0.012	0.87
S5	2.596	0.031	−1.141	0.013	0.96

**Table 2.** Calibration constants of Equation (4) from the direct calibration (reference gas: air, outer matrix: air with added CO_2_).

**Sensor**	*k*_0_ **[%_vol_]**	*δk*_0_ **[%_vol_]**	*k*_1_ **[%_vol_ s/mbar]**	*δk*_1_ **[%_vol_ s/mbar]**	*δp*_*CO*_2__ **[ppm]**
S1	0.0187	0.0011	21.105	0.026	210
S2	0.0055	0.0013	20.695	0.030	243
S3	−0.0009	0.0013	20.848	0.030	247
S4	0.0151	0.0011	20.403	0.026	212
S5	0.0099	0.0013	20.696	0.029	234

**Table 3.** Calibration constants of Equation (5) from the inverse calibration method (reference gas: air with added CO_2_, outer matrix: air).

**Sensor**	$k_{0}^{\mathrm{inv}}$ **[%_vol_]**	$\delta k_{0}^{\mathrm{inv}}$ **[%_vol_]**	$k_{1}^{\mathrm{inv}}$ **[%_vol_ s/mbar]**	$\delta k_{1}^{\mathrm{inv}}$ **[%_vol_ s/mbar]**	*δp*_*CO*_2__ **[ppm]**
S1	0.0227	0.0019	−21.701	0.053	269
S2	0.0231	0.0020	−22.086	0.054	272
S3	0.0231	0.0018	−21.500	0.049	253
S4	0.0221	0.0018	−21.454	0.047	242
S5	0.0156	0.0018	−21.452	0.047	246

**Table 4.** Concentration-dependent standard deviations (s [ppm]) for registration of the concentrations C_1_–C_4_ (in Figure 3) observed by line-sensors (S1–S5) and the reference (Ref). The line-sequence was arranged with respect to increasing concentrations C_1_ ≈ C_4_ < C_2_ < C_3_.

$p_{{\mathrm{CO}}_{2}}^{a}$	**s(S1)**	**s(S2)**	**s(S3)**	**s(S4)**	**s(S5)**	**s(Ref)**
C_1_	182	180	181	188	130	105
C_4_	197	194	239	184	183	110
C_2_	133	169	161	134	108	250
C_3_	120	283	219	188	117	369

**Table 5.** Calibration constants from the inverse calibration method (correlation coefficient > 0.996) against the unknown background concentration (reference gas: air with added CO_2_, outer matrix: air with added CO_2_-background).

**Sensor**	$k_{1}^{\mathrm{inv}}+p_{{\mathrm{CO}}_{2}}^{a}$ **[%_vol_]**	$\delta k_{0}^{\mathrm{inv}}$ **[%_vol_]**	$k_{1}^{\mathrm{inv}}$ **[%_vol_ s/mbar]**	$\delta k_{1}^{\mathrm{inv}}$ **[%_vol_ s/mbar]**	*δp_CO2_* **[ppm]**
S1	0.9140	0.0029	−22.415	0.095	338
S2	0.9262	0.0034	−22.660	0.114	404
S3	0.9152	0.0032	−22.315	0.104	377
S4	0.9246	0.0032	−22.238	0.105	379
S5	0.9039	0.0032	−21.942	0.103	374

**Table 6.** Mean background concentrations from inverse calibration.

**Sensor**	$p_{{\mathrm{CO}}_{2}}^{a}$ **[%_vol_] (Test I)**	$p_{{\mathrm{CO}}_{2}}^{a}$ **[%_vol_] (Test II)**
S1	0.8831	0.8913
S2	0.8963	0.9031
S3	0.8826	0.8921
S4	0.8930	0.9025
S5	0.8827	0.8883
